# Inaccuracies of the ISO 11731 Method for Environmental Validation of *Legionella* in Building Water Systems: Opportunities to Improve Sensitivity and Detect Viable but Non-Culturable *Legionella*

**DOI:** 10.3390/microorganisms11010094

**Published:** 2022-12-30

**Authors:** Leah P. Wickenberg, Katherine E. Fisher, Melissa F. Cain, William F. McCoy

**Affiliations:** Phigenics Research and Innovation Team, 1664 N. Virginia Street, MS 0525, Reno, NV 89557, USA

**Keywords:** *Legionella*, viable but non culturable, water management, monochloramine

## Abstract

Current environmental diagnostics for the detection of *Legionella* fail to detect viable but non-culturable *Legionella*, have sensitivity limitations and are time-consuming (10–14 days to results). The objective of this study was to compare *Legionella* detection results between the standard ISO 11731 and an innovative *Legionella* detection method that utilizes a hybrid methodology of traditional microbiology and molecular detection. In this study, four hundred and seventy-six (476) potable building water samples were analyzed with ISO 11731 and the novel method in parallel. Of the 476 total samples that were tested, a discrepancy of 21% was observed when comparing the ISO 11731 method to the novel method. Separating the samples based on hazard control methods yielded a 15.4% discrepancy for chlorinated systems (*n* = 284) and a 29% discrepancy for monochloraminated systems (*n* = 192). The data presented here conclusively show inaccuracies in environmental validation for building water systems based on results returned by the standard ISO 11731 method. This is especially evident in systems primarily disinfected with monochloramines. Overall, these data highlight the need for new and innovative methods to overcome the inaccuracies of the traditional ISO 11731 spread plates to prevent disease and injury caused by *Legionella*.

## 1. Introduction

Opportunistic waterborne pathogens in building water systems are a serious cause for concern. These include many species of *Legionella,* such as *Legionella pneumophila, Legionella longbeachae, Legionella anisa,* and *Legionella micdadei* [1]. *Legionella* infections occur via the inhalation of aerosolized water droplets containing *Legionella* bacteria. Inhalation most often occurs in the built environment [2], and the risk of inhalation is greatly increased during construction activities [3]. Infections caused by these organisms often go unreported, but there are an estimated 13,000 infections annually in the United States for *Legionella* spp. [4]. Legionnaires’ disease presents distinctly as pneumonia, usually requires hospitalization, and has an estimated fatality rate of approximately 10% [5].

Current detection methods for *Legionella* from environmental and clinical samples are time-consuming (10–14 days for results) and labor-intensive [6,7]. They also contain extensive confirmation steps that can be subjective. Furthermore, the sample collection volumes (100–1000 mL), and pre-sample flush times (2 min) have not been standardized across reference methods for *Legionella* testing [8,9]. An additional factor to consider is the relative risk associated with different sample volumes and limits of detection (LOD). A 2018 paper states that it may not be necessary to filter more than 100 mL of water for environmental validation due to the lack of risk when detections are <10 CFU/mL [10]. In addition to these well-documented limitations, the traditional spread plate methods cannot detect alternative forms of *Legionella*, such as viable but non-culturable (VBNC).

Conventional methods rely on the typical definition of “viable” as being the ability to produce colonies of bacteria (progeny) on the surface of solid growth media, as described in the ISO 11731 method [11]. Many bacterial genera and species have been found to exist in the VBNC state since its discovery 30 years ago [12]. Specifically, *Legionella* is both metabolically active [13] and infective [14], in the VBNC state. Loss of culturability results in false identification and underestimation of the viable cells in an environmental sample, but it does not indicate a complete loss of viability or infectivity. These inaccuracies can pose a risk to public health because VBNC cells have demonstrated pathogenic properties [12,15]. The induction of the VBNC state may be a result of exposure to various stresses [16,17,18]. These include exposure to non-lethal temperatures, starvation, and chemical stresses. VBNC cells can be resuscitated naturally under specific environmental conditions [18]. They can also be resuscitated in the laboratory by co-culture with natural protozoan hosts [19]. The limitations mentioned above have increased the demand for a more useful definition of the word “viable” and a rapid way to measure it.

There are four non-conventional approaches to detecting the viability of microbial cells that do not rely on traditional microbiological techniques. These four test methods are described as: viability PCR (vPCR), measurement of the cellular membrane potential, measurement of the concentration of adenosine triphosphate (ATP), and enzymatic kinetic measurements [13]. Although these methods are valid indicators of viability, they can also detect VBNC cells, leading to a poor correlation with traditional culture methods [19,20]. While this poor correlation to traditional spread plates could indicate that these methods have a higher sensitivity and accuracy, overall limitations of these test methods (membrane potential, ATP, and kinetic) result in impractical application for routine validation testing of building water systems. Of the four methods, viability PCR methods (vPCR) are being used more often in environmental testing. This method relies on the ability of intercalating DNA dyes to penetrate the cell and render the DNA useless for PCR [21,22]. Research has shown that typical concentrations of chemical disinfectants do not cause sufficient damage to the cellular membrane to allow this method to distinguish between viable and non-viable cells [23,24].

Alternative culture methods that attempt to overcome limitations of the ISO 11731 spread plate method (i.e., extended turnaround times) are outlined in the recently published ASHRAE Guideline 12-2020: Managing the Risks of Legionellosis Associated with Building Water Systems [25]. This guidance states that in-field inoculation devices are an acceptable variation for culture-based methods in Appendix C, Section 3.1a. While these in-field inoculation devices can reduce the turnaround time, the methods present sensitivity limitations for potable water samples compared to ISO 11731 spread plate methods. However, a 2012 Water Research study documents that using in-field inoculation devices could eliminate the effects of holding time on water samples, leading to more accurate results from building water system samples [26]. This alternative culture approach and the abovementioned non-conventional viability approaches are scientifically valid; however, they still have limitations. The main limitations for the more practical methods (in-field sampling and vPCR) are that VBNC *Legionella* can cause false negative (in-field sampling) or false positive (vPCR) results.

The data presented here describes a large-scale field study that highlights the deficiencies of the ISO 11731 method and those present with the alternative methods mentioned above. A novel method, VIABLE (Viability Identification Assay by *Legionella* Enrichment), was used to identify and overcome the ISO 11731 inaccuracies. This was achieved by testing 476 real-world water samples with both methods. These inaccuracies are especially evident in building water systems primarily disinfected with monochloramines, where there is a discrepancy of up to 29% when comparing ISO 11731 to VIABLE. This is due to the possible presence of VBNC *Legionella* in these systems. In summary, these data reinforce the need for a practical and more sensitive way to detect all forms of viable *Legionella* in a building water system and presents a novel method that can do so.

## 2. Materials and Methods

### 2.1. VIABLE (Viability Identification Assay by Legionella Enrichment)

**Figure 1:**
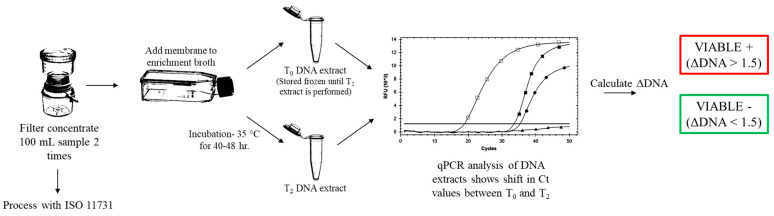
Flow chart showing the sample analysis with VIABLE and ISO 11731.

Water samples and engineered water samples (100 mL) were filter concentrated using 0.2 μm, 47 mm polycarbonate track-etched filter membranes (GE Healthcare, Chicago, IL, USA). The membrane was aseptically transferred to a 25-mL Nunc Cell Culture EasYFlask (Fisher Scientific, Hampton, NH, USA) containing 10 mL of EB7 broth (Phigenics, LLC, Warrenville, IL, USA). Flasks were gently swirled by hand for 30 s to resuspend concentrated bacteria. A 2.0-mL aliquot of the suspension was removed and used for the T_0_ timepoint DNA extraction. Flasks were then placed in the incubator at 35 °C with shaking at 50 rpm for up to 48 h. DNA extractions were taken at timepoints T_0_ = 0 h, and T_2_ = 40–48 h. These timepoints were used to assess the change in concentration of DNA over time (ΔDNA), which is an indicator of microbial growth. DNA extracts were analyzed using a commercially available real-time polymerase chain reaction (qPCR) kit specific to the *Legionella* genus (Bio-Rad, Hercules, CA, USA). The genomic units for the extracts were not calculated. The T_0_ DNA extracts were stored at −20 °C for 48 h. Both T_0_ and T_2_ timepoint extracts were analyzed at the same time with qPCR to eliminate any inter-run variations that may impact the analysis of the ΔDNA. A graphical representation of the method can be seen in Figure 1.

### 2.2. DNA Extraction

The DNA extraction utilized P.U.R.E. (Phigenics Ultra-Rapid DNA Extraction). This is a proprietary method developed and validated by Phigenics, LLC (Warrenville, IL, USA).

### 2.3. qPCR Detection

Real-time PCR was used to detect a change in the concentration of DNA found in the liquid cultures of the VIABLE assay. This was performed using a commercially available qPCR kit for *Legionella* species as per the manufacturer’s instructions on a CFX96 Deep Well Touch thermocycler (Bio-Rad, Hercules, CA, USA). DNA extracts were analyzed to determine if there were changes in the cycle threshold (Ct) values during the incubation. T_2_ Ct values were subtracted from the T_0_ Ct values to yield the ΔDNA result. Ct values were determined by the CFX IDE software provided with the thermocycler. Timepoint extracts from each experiment were analyzed at the same time using external and internal controls. External controls included both a positive and a negative control provided by the manufacturer, a sterility check control, and a reagent control. The sterility check control consisted of a sterile broth control that was extracted simultaneously, using the same protocol as the experimental samples. The reagent control is introduced at the beginning of P.U.R.E. and demonstrates that no contamination was introduced during the DNA extraction. The internal control was included in the buffer portion of the qPCR kit.

### 2.4. ISO 11731:2017 Spread Plate Method

Analysis of the VIABLE method was compared to the standard culture method using ISO 11731:2017 with the following modifications: samples were not pretreated before analysis and were plated only on GVPC agar. In short, samples were concentrated exactly as described above and 100 μL aliquots of the concentrated sample were spread-plated on GVPC agar (Phigenics, LLC, Fayetteville, AR, USA). Samples were incubated at 35 °C for 10 days. This was completed for both timepoints collected for DNA extraction to show a correlation between growth in the liquid broth cultures, detected by ΔDNA analysis and the conventional culture media. Suspect *Legionella* colonies were confirmed via cysteine auxotrophy and latex agglutination as recommended by the standard method.

### 2.5. Statistical Analysis

McNemar’s Chi Squared between VIABLE and ISO was analyzed. The outcome was considered positive if growth was detected. The 2 × 2 contingency table was created and analyzed here: http://vassarstats.net/propcorr.html (accessed on 8 December 2022). The Odds Ratio, *p*-value, and 95% confidence interval were also calculated. The null hypothesis is that there is no difference between ISO 11731 and VIABLE. The alternate hypothesis is that there is a difference if *p* < 0.05. Table 1 shows an example of the 2 × 2 contingency table.

### 2.6. Field Testing

Four hundred seventy-six (476) potable water samples were collected from various building water systems across the United States. Samples were analyzed at Phigenics Analytical Service Laboratories (PASL) in Warrenville, IL, USA and Fayetteville, AR, USA, and Phigenics Research and Innovation Team Laboratory (phiRIT, Reno, NV, USA). One hundred milliliters of water were collected in sterile containers containing 10 mg of sodium thiosulfate and shipped overnight to the desired location at ambient temperature. Samples were analyzed using the above methods of VIABLE and ISO 11731:2017 in tandem. 

### 2.7. Co-Culture with Amoebae

*Acanthamoeba castellanii*, generously provided by Dr. Howard A. Shuman (University of Chicago, Chicago, IL, USA), were cultured in pyruvate, yeast extract, and glucose broth (PYG). For the co-culture experiment, the amoebae were counted using a hemocytometer with a Fuchs Rosenthal ruling and the manufacturer’s instructions for sample volume and calculations (Thomas Scientific, Swedesboro, NJ, USA). The cells were visualized on a Leica DM 1000 LED microscope (Nevada Center for Applied Research, Reno, NV, USA) and diluted to 10^3^–10^4^ cells/mL in PYG. Twenty-eight potable water samples from a chloraminated water system were filter-concentrated (100 mL) onto a 0.2-µm membrane. The membrane was acid-treated on the filter unit for five minutes with 16 mM Glycine HCl (pH 2.2) to decrease the number of competing bacteria. The membrane was rinsed with 20 mL of PBS while on the vacuum filter unit. For samples 17–28 a mixture of Glycine, Vancomycin, Polymyxin-B, and Cycloheximide (GVPC) was added to the PYG to decrease the number of competing bacteria. The final concentrations of each GVPC component were 1.01 mg/mL, 0.338 μg/mL, 27 IU/mL, and 0.027 mg/mL, respectively. The filter membrane was then aseptically transferred to 25-mL Nunc Cell Culture EasYFlask (Fisher Scientific, Hampton, NH, USA) containing 10 mL of PYG with 10^3^–10^4^ cells/mL *A. castellanii.* This process was repeated to analyze the samples using the VIABLE protocol simultaneously. T_0_ and T_2_ timepoints were analyzed by DNA extraction and qPCR. ΔDNA analysis was performed for each sample as stated above. Statistical analysis of this sample set was performed as described above in Section 2.5. The null hypothesis is that there is a difference between co-culture with amoeba and VIABLE or ISO 11731. The alternate hypothesis is that there is no difference between co-culture with amoeba and VIABLE or ISO 11731.

## 3. Results

### 3.1. Field Testing

**Figure 2:**
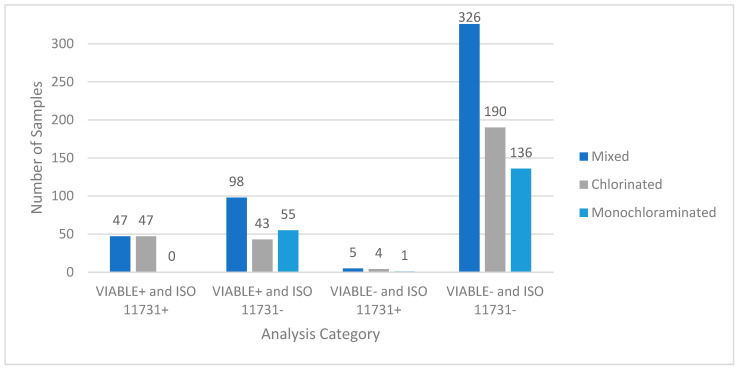
Field test results comparing ISO 11731 to VIABLE. A total of 476 potable water samples from building water systems were tested by VIABLE and ISO 11731:2017. These data indicate that VIABLE can reliably detect viable *Legionella* in a building water system. It also indicates that VIABLE is more sensitive than the ISO 11731:2017 method because of the 98 samples that were positive for VIABLE and negative on the spread plates. The five VIABLE-negative, culture-positive (invalid) results ranged from <1–30 CFU/mL on the culture plates. Statistical analysis of the dataset can be seen in Table 1 and Table 2. The sample is also shown stratified by the HC method in this figure.

Figure 2 depicts data from a large-scale field study conducted on potable and non-potable water systems across the United States. Out of the 476 samples, 326 (68%) were negative for viable *Legionella* for both methods, 47 (10%) were positive for both methods, 98 (21%) were positive for VIABLE and negative for ISO 11731), and 5 (1%) were negative for VIABLE and positive for ISO 11731. Table 2 shows the statistical analysis used to compare the two methods. The ISO 11731 method was used as the comparison method to calculate the statistics because it is the accepted standard method. The PPV was 32.4%, and the NPV was 98.5% for the combined dataset. There is a high discrepancy rate between ISO 11731 detections and VIABLE detections. To determine if this discrepancy was significant, the McNemar Chi-Squared test was used. Statistical results are shown in Table 3. There is a significant difference between VIABLE and ISO (*p* < 0.05), indicating that the null hypothesis can be rejected, and the alternate hypothesis accepted. To determine a cause for this discrepancy, the sample set was separated by the hazard control (HC) method between chlorine and monochloramine disinfectants based on what was received at the building.

### 3.2. Hazard Control: Chlorine

Two hundred and eighty-four (284) samples were taken from facilities that utilize chlorine as the HC method (Figure 2). One hundred and ninety samples were negative for both methods (66.9%), 47 (16.5%) samples were positive for both methods, 43 (15.4%) samples were positive for VIABLE and negative for ISO 11731, and 4 (1.4%) samples were negative for VIABLE and positive for ISO 11731. Statistical analysis for the chlorinated samples can be seen in Table 3. Percent positivity for VIABLE and ISO 11731 increased in chlorinated systems (31.7% and 18%, respectively) compared to the combined set in Figure 1. The PPV of VIABLE increased (52.2% vs. 37.4%) for chlorinated samples only, while NPV decreased slightly. Table 3 shows that there is a significant difference (*p* < 0.05) between VIABLE and ISO 11731 for samples where the HC method is chlorine. The null hypothesis is rejected for this dataset, and the alternate hypothesis is accepted.

### 3.3. Hazard Control: Monochloramine

Systems with monochloramine as the HC method showed distinctly different results. Figure 1 shows the results of the 192 samples taken from monochloraminated systems. There were 136 (71%) samples that were negative for both methods, 0 samples that were positive for both methods, 55 (29%) samples that were positive for VIABLE and negative for ISO 11731, and 1 sample that was negative for VIABLE and positive for ISO 11731. Statistical analysis is shown in Table 3. The ISO 11731 positivity percentage drops to 0.5%, whereas the VIABLE positivity percentage (28.5%) remains similar to the two other sample sets. Sensitivity and NPV for VIABLE also remained similar (98.2% and 99.27%, respectively). The PPV was 0% due to the lack of ISO 11731 positive samples. These results indicated that VBNC *Legionella* were present in the monochloraminated water samples. To test this hypothesis a second set of samples were taken from a monochloraminated system and tested with VIABLE, co-culture with *A. castellanii*, and ISO 11731. Table 3 shows a significant difference (*p* < 0.05) between VIABLE and ISO 11731 for samples where the HC method is monochloramine. The null hypothesis is rejected for this dataset and the alternate hypothesis is accepted.

### 3.4. Confirmation of VBNC Legionella Using Amoeba Co-Culture

Twenty-eight (28) samples were taken from a monochloraminated water system that were analyzed using VIABLE, amoeba co-culture, and ISO 11731. No positive samples were recorded using the ISO 11731 method. There were 15 (53.4%) samples that were negative for all three methods, 7 (25%) samples that were positive for both VIABLE and amoeba co-culture, 4 (14.3%) samples were positive for VIABLE and negative for amoeba co-culture, and 2 (7%) samples were negative for VIABLE and positive for amoeba co-culture (Table 4). Statistical analysis comparing VIABLE to co-culture is shown in Table 4. Co-culture results were set to true. The percent positivity for VIABLE and co-culture were similar (39.3% and 32.1%, respectively). The sensitivity, PPV, and NPV were 77.8%, 63.6%, and 88.2%, respectively. Considering the 15 samples that were negative for all three methods, these data indicate that the ISO 11731 method returned false-negative results for 46.4% of samples from this monochloraminated system. These data indicate that there is not a significant difference between amoeba co-culture and VIABLE, therefore, the null hypothesis cannot be rejected. However, there is a significant difference (*p* < 0.05) between ISO 11731 and amoeba co-culture. Therefore, the alternate hypothesis is accepted. The 2 × 2 contingency table used to complete the statistical analysis is shown in Table 5.

## 4. Discussion

The discrepancy between non-detectable ISO 11731 cultures and VIABLE positive detections presents challenges for teams working to ensure an effective Water Management Program. The difference between the two methods can be attributed to two factors: a lack of sensitivity in systems with chlorine as the HC method and the presence of VBNC *Legionella* in systems with monochloramine as the HC method. Fifteen percent (43 samples) of the samples from water systems disinfected with chlorine were detected by VIABLE but not by ISO 11731. This indicates that 43 water samples were found to be non-detectable using the standard ISO 11731 method, however the VIABLE method reported detectable *Legionella*. Based on the presence of viable *Legionella* at these distal locations in the building water system, using stand-alone ISO 11731 results for validation purposes may present missed opportunities to optimize water management program controls. Furthermore, samples with non-detectable ISO 11371 results that are positive via the VIABLE method present an increased risk of legionellosis if exposure occurs with high-risk individuals.

There are many different proposed methods to increase the sensitivity of the ISO 11731 method. The 2017 version of the ISO method suggests plating on multiple media types (BCYE, GVPC, MWY, etc.) to ensure recovery of all Legionella in a sample [11]. This can lead to extended sample processing times and elevated costs [27]. A higher sensitivity may also be achieved by sampling larger volumes of water to increase the concentration factor of the sample. The CDC recommends collecting 1000 mL of water during an outbreak investigation (https://www.cdc.gov/legionella/downloads/cdc-sampling-procedure.pdf (accessed on 15 March 2020). However, routine shipment of 1 L water samples is cumbersome and costly. PCR has also been more sensitive than traditional culture-based testing [28]. However, PCR is not a valid indicator of viability. Using a combination of culture enrichment and PCR detection eliminates the abovementioned drawbacks.

The data presented here show numerous discrepancies when using the ISO 11731 method to analyze water systems disinfected by monochloramines. Research has shown that disinfection with monochloramines can induce the VBNC state in *Legionella* [1], [2] and that these bacteria are still metabolically active [3] and infective [4]; Within the current dataset, 28.6% of these samples were VIABLE positive ISO 11731 negative samples (Figure 1). It is worth noting that the single positive result from the ISO method was a VIABLE false negative. There was a large amount of competing microbiota in this sample which is the most probable reason for the false negative VIABLE result. The *Legionella* isolate was tested with the VIABLE method as a pure culture (data not shown) to test this hypothesis. It returned positive results indicating that the specific strain of *Legionella* was able to grow without competition using the VIABLE method. The positive samples detected by VIABLE and amoeba co-culture represent 28.6% increase in exposure points to potentially infective *Legionella.* These potential exposure sites were not detected using the conventional ISO 11731 method. Of the 10 systems tested in this study with monochloramines as the HC method, VIABLE reported an average positivity percentage of 32.9% (data not shown) for each system. Only one sample (discussed above) yielded a positive ISO 11731 culture, indicating that almost all 32.9% were inaccurately reported as non-detectable results in these systems. These data also suggest that these inaccurate ISO 11731 results are due to the presence of VBNC *Legionella* because the positivity percentage for VIABLE is comparable to that of the field testing results (32.9% vs. 31%, respectively) and to that of the samples primarily disinfected with chlorine (32.9% vs. 32%, respectively). VBNC *Legionella* was confirmed by testing one of the 10 systems with amoeba co-culture, VIABLE, and ISO 11731 in parallel.

The novel method, VIABLE, performed just as well as if not better than co-culture with protozoa (Table 3). Seven of the positive samples were detected by both methods (25%). Four samples were detected by VIABLE and not by co-culture (14%). This could be due to the increased sensitivity of the VIABLE assay. The results of the two samples that were positive for co-culture but negative for VIABLE could be due to high concentrations of competing microbiota. Theoretically, a single cell of *Legionella* could multiply within the 48 h to detectable levels by VIABLE. When working with co-culture, the method is dependent on the natural cycle of host infection which may or may not happen within the 48 h time this assay was performed. This is the principal limitation of utilizing co-culture techniques for routine validation testing. These data support the hypothesis that VIABLE overcomes the co-culture limitation while retaining the ability to resuscitate VBNC *Legionella* efficiently. Though this sample set is limited, the data suggests that VIABLE may be a faster and more efficient way of resuscitating VBNC *Legionella.* The combination of VIABLE and co-culture positive results from this sample set indicates a 46% (13/28) false negative percentage for the ISO 11731 spread plate method. The data support that these false negative results are due to VBNC *Legionella* in the system.

Overall, these data indicate that the ISO 11731 method can yield inaccurate results when used to analyze building water systems, notably when the population of VBNC *Legionella* are present or when low LOD testing is required. Utilizing innovative *Legionella* diagnostics during outbreak investigations and routine validation testing can further prevent disease and injury. Developing and implementing a water management program aligned with ASHRAE Standard 188 reduces the risk of disease and/or injury associated with the building water system. ANSI/ASHRAE Standard 188-2021 Section 6.2.8 defines validation as confirmation that “the Program, when implemented as designed, controls the hazardous conditions throughout the building water systems.” The limitation of current test methods could impact the validation strategy because they cannot detect all infective forms of *Legionella* bacteria. The decisions made from traditional spread plate test results could lead to a less defensible position for water management teams. These test results may not be sensitive enough to confirm the effectiveness of implemented hazard control leading to a WMP that is not optimized for long-term performance and disease prevention.

## 5. Limitations

This study compared VIABLE to one of the ISO 11731 media options, GVPC. Though Scaturro et al. 2020 showed that there was not a significant difference between the recovery of *Legionella* on BCYE and GVPC, it is worth noting that other sources have shown a difference in recovery with regard to certain *Legionella* species [29]. In the future, the ISO 11731 inaccuracy evaluation should also include samples that are tested with BCYE media. Wide confidence intervals are also a limitation of this study. This can indicate that there is a high degree of variability within the samples. The authors want to investigate this statistical finding further, but it was not in the scope of this study to investigate wide confidence intervals.

## 6. Conclusions

Overall, the data presented here highlight the need to innovate novel methods for *Legionella* detection in environmental samples to improve the effectiveness of validation testing within a WMP. As more information about the VBNC *Legionella* state becomes available, the need to accurately detect these alternative forms has increased. Lastly, VBNC *Legionella* must be considered when developing a water management program, especially for systems where the primary mode of disinfection is monochloramine. VIABLE is a novel method that presents a practical way for a water management team to implement a successful control strategy for water quality and safety by detecting VBNC *Legionella*.

## Figures and Tables

**Table 1:** Example 2 × 2 contingency table used to compute statistics.

	ISO 11731 +	ISO 11731 −
VIABLE +	47	98
VIABLE −	5	326

**Table 2:** Statistical analysis of field study samples. Samples were analyzed for percent positivity, positive predicative value, and negative predictive value. ISO 11731 method values differed between the different HC methods. VIABLE values stayed consistent between the different HC methods.

Hazard Control Method	Total # of Samples	ISO 11731 Positive (%)	VIABLE Positive (%)	Positive Predictive Value (%)	Negative Predictive Value (%)
Chlorine	284	18.0	31.7	52.2	97.9
Monochloramines	193	0.5	28.5	0.0	99.3
Combined Data	477	10.9	30.4	32.4	98.5

**Table 3:** McNemar Chi Squared analysis of field study samples. Samples were analyzed using the McNemar Chi Squared test designed to calculate significant differences between the methods. For all categories there was a significant difference between ISO 11731 and VIABLE (*p* < 0.05).

Hazard Control Method	Total # of Samples	McNemar Test Result (Two Tail)	Odds Ratio	95% Confidence Interval (Lower Limit, Upper Limit)
Chlorine	284	<0.01	11.75	(4.23, 32.61)
Monochloramines	193	<0.01	55.00	(7.61, 397.45)
Combined Data	476	<0.01	9.40	(3.74, 23.63)

**Table 4:** Statistical analysis of samples tested with co-culture, VIABLE, and ISO 11731. McNemar Chi Squared was used to determine if there was a significant difference between detections between the three methods. The odds ratio and 95% Confidence Interval were calculated for VIABLE. PPV and NPV were calculated for VIABLE and ISO 11731. The data indicate that there is a significant difference between ISO 11731 and co-culture with amoeba. There is not a significant difference between VIABLE and co-culture with amoeba.

Method vs. Co-Culture	Total # of Samples	McNemar Test Result (Two Tail)	Odds Ratio	95% Confidence Interval (Lower Limit, Upper Limit)	Positive Predictive Value	Negative Predictive Value
ISO	28	<0.01	N/A *	N/A *	0.0%	32.1%
VIABLE	28	0.69	2.00	(0.37, 10.92)	63.6%	88.2%

*—Odds ratio and 95% confidence interval were not able to be calculated due to lack of positive results.

**Table 5:** VIABLE results in comparison to amoeba co-culture. A total of 28 chloraminated potable water samples were tested with VIABLE and with co-culture with *A. castellanii*. The 2 × 2 contingency table used to calculate the statistics in Table 3 is shown here. Samples were analyzed using a ΔDNA cutoff value of ≥0.5. All tested samples were negative using the ISO 11731:2017 method. These data show that VIABLE and co-culture with amoeba can resuscitate and detect VBNC *Legionella*.

	Co-Culture +	Co-Culture −
VIABLE +	7	4
VIABLE −	2	15

## Data Availability

The data presented in this study are available on request from the corresponding author. The data are not publicly available due to privacy.
